# Identification of a binding pocket of letermovir in the terminase subunit pUL56 of human cytomegalovirus

**DOI:** 10.1038/s41598-025-94809-1

**Published:** 2025-03-25

**Authors:** Lukas M. Kmetsch, Hans Tietze, Elke Bogner

**Affiliations:** Institute of Virology, Charité - Universitätsmedizin Berlin, Freie Universität Berlin and Humboldt-Universität Zu Berlin, 10117 Berlin, Germany

**Keywords:** Human cytomegalovirus, Terminase, Letermovir, Binding pocket, Computational biology and bioinformatics, Drug discovery, Microbiology

## Abstract

A key step in replication of human cytomegalovirus (HCMV) is the generation and packaging of unit-length genomes into preformed capsids. The enzymes involved in this process are viral terminases. The HCMV terminase consists of two subunits, the ATPase pUL56 and the nuclease pUL89. A potential third component, pUL51, has been proposed. Letermovir is the first terminase inhibitor available for HCMV prophylaxis to allogenic hematopoietic stem cell recipients. However, mutations in the HCMV terminase subunit pUL56 and, to a lesser extent, in pUL89 or pUL51 lead to resistance. Here we focused on the drug target area in the terminase subunit pUL56. To gain further structural insights into the putative binding site of letermovir, in silico analysis of the structure was performed using Phyre2 and SwissDock. For our analysis, we used three of the most frequent mutations during letermovir treatment, C325F, C325Y and C325W. We demonstrated that all variants have a pronounced cavity reduction, leading to the letermovir binding conformations being “pushed-out” of the binding pocket. This results in a changed distribution of the Gibbs free binding energy. To circumvent the absolute resistance of C325 mutations a further modification of letermovir might solve the problem and leads to optimizing drug targeting capacity.

## Introduction

Human cytomegalovirus (HCMV) is classified within the subfamily Betaherpesvirinae, in the Herpesviridae family. HCMV is a ubiquitous opportunistic pathogen and a major human pathogen, characterized by a specific host range and a prolonged replication cycle in host-specific cell cultures^1,2^. Although HCMV infection is usually asymptomatic in most individuals, it has been shown to cause severe disease with significant morbidity and mortality in newborns and immunocompromised patients such as organ recipients and AIDS patients^2–4^. The virus persists by establishing a lifelong latent infection, evading recognition by the immune system^3^.

Maturational events of HCMV DNA replication and capsid assembly require cleavage of concatenated DNA to genome length, followed by insertion into preformed capsids. Since this process is absent in mammalian cells, compounds targeting this unique, virus-specific process should promise higher efficacy and greater safety than currently approved drugs.

Herpesvirus replication occurs as rolling circle replication leading to concatemers, head-to-tail linked unit length genomes. The genome contains the so-called *a-sequence* that is located at both terminal repeats and in opposite orientation between the L- and S-segment^4,5^. In the *a-sequence*, the specific motifs pac1 and pac2 are located. These motifs are necessary for cleavage of concatemers into unit length genomes and packaging them into preformed capsids^6^. Enzymes involved in the viral DNA packaging process are responsible for site-specific duplex nicking and insertion of DNA into procapsids^7,8^. Terminases were first described for double-stranded DNA bacteriophages and are highly conserved in many dsDNA viruses, e.g., herpesviruses and adenoviruses. Terminases, which are multifunctional heterooligomers, are responsible for packaging the viral DNA into procapsids to form infectious particles. As such, different subunits catalyses ATP-dependent translocation into preformed capsids and the cleavage of DNA concatemers into unit-length genomes^9–12^. The following six steps are involved in this process: (i) The recognition of viral DNA by a specific protein able to (ii) bind DNA at specific sequence motifs (packaging signals, e.g., pac1 and pac2), (iii) translocation of the DNA–protein complex to the procapsid, (iv) interaction of the DNA–protein complex with the portal protein, (v) import of one unit-length genome into a capsid involving ATP hydrolysis by one terminase subunit, and (vi) completion of the packaging process by cleavage of excess DNA (two strand nicking). The HCMV terminase complex includes two subunits, the ATPase pUL56 and the nuclease pUL89^13–16^. A potential third component pUL51 has been proposed^17,18^. Letermovir is the first terminase inhibitor available for HCMV prophylaxis following allogenic hematopoietic stem cell transplantation^19^. It has been hypothesized that letermovir prevents the formation of infectious viral particles by targeting the pUL56 subunit^20,21^. Goldner et al.^19^ demonstrated that letermovir prevents replication without inhibition of viral DNA and protein synthesis. This is in line with other terminase inhibitors, which, although not fully blocking all viral DNA processing steps, still prevent the formation of functional genome structures^22,23^. In addition, no reports are available demonstrating a direct binding of letermovir to pUL56. Overall, the molecular mechanism by which letermovir exerts its antiviral effect has not been determined. Mutations in the HCMV terminase subunit pUL56 and, to a lesser extent, in pUL89 and pUL51 may also drive resistance^24^. In contrast to pUL56 all mutations of pUL89 and pUL51 confer low level resistance, therefore it is unlikely that they cause clinical concerns^19,24^. Several amino acids substitutions discovered in pUL56 (C325Y/F) confer high levels of resistance and lead to manifestation of clinical resistance. Noteworthy, mutants with high resistance against letermovir show no growth impairment^25,26^. Therefore, it is reasonable that these pUL56 mutations have no effect on the formation or stability of the terminase complex. In this study, analyses on the mutants pUL56 C325F/W/Y as well as V236M were performed, and a potential binding pocket of letermovir in the terminase subunit pUL56 was determined.

## Results

### Determination of the 3D structure of pUL56

**Fig. 1:**
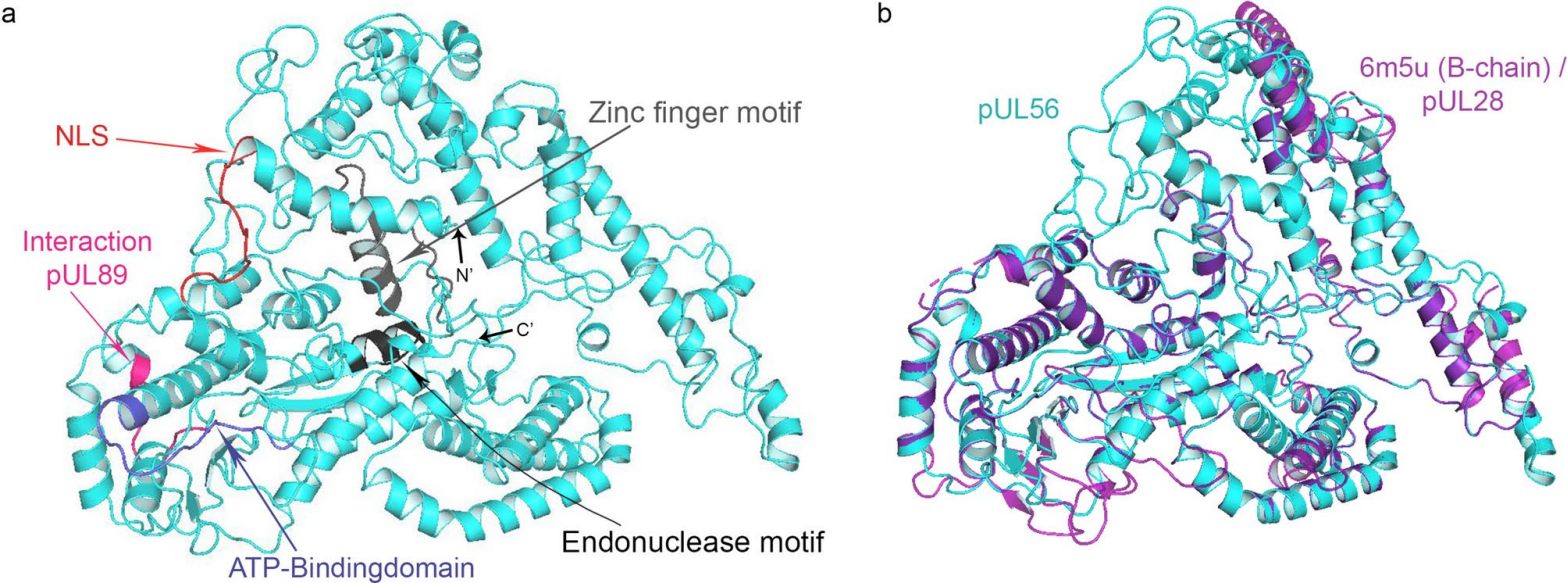
Structural prediction of full-length pUL56. (**a**) The ribbon representation of pUL56 model with different motifs. (**b**) Structural model of pUL56 at pH 7.2 (cyan) and the overlay with the homologous small terminase subunit of HSV-1 strain 17, pUL28 (magenta).

To identify potential binding sites of letermovir, in silico analysis of the pUL56 structure was performed using Phyre2^27^ in intensive mode. The analysis took several structures of homologous proteins into account. The small terminase (Ter^s^) subunit pUL56 showed a unique structure with many domains. Included are the nuclear localization signal^28^, the ATP binding domain^29,30^, and the interaction domain with pUL89^31^. In addition, the proposed endonuclease and zinc finger motifs are included^32^. A scheme of the predicted structure of pUL56 monomer is shown in Fig. 1a. Interestingly, the newly generated structure of pUL56 is highly homologous to the crystal structure of the small terminase subunit of Herpes simplex virus type 1 (HSV-1; strain 17), pUL28 (PDB: 6m5u, B chain). Phyre2 reports the confidence for homology between these two subunits as 100%. The graphical alignment of both Ter^S^ proteins is shown in Fig. 1b. Furthermore, Phyre2 found structural homologies with human topoisomerases (85–90% confidence homology). These enzymes have similar function to viral terminases. They initiate double strand cuts and have the ability to hydrolyse ATP. Our generated, monomeric 3D structure of pUL56 has a high confidence of the model and homology to proteins with similar functions. This structure was used for further in silico analysis.

### Mutations of pUL56 induced by letermovir

**Fig. 2:**
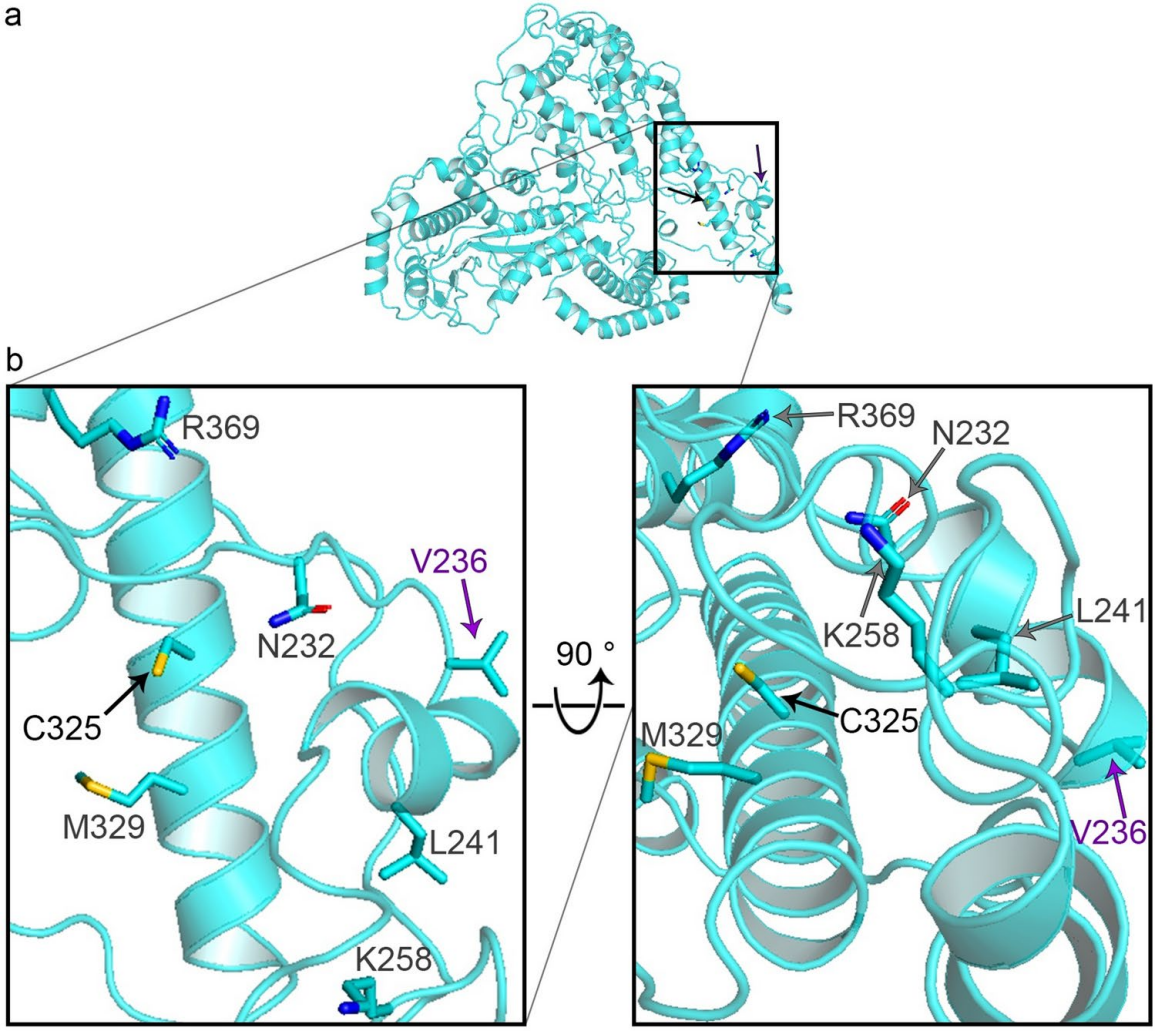
Mutations of pUL56 induced by letermovir. (**a**) Localization of most reported mutants. (**b**) Residuals of most prominent mutants studied in this paper: C325 (black arrow) and V236 (magenta arrow). Oxygen-red, Nitrogen-blue, Sulfur-orange

**Fig. 3:**
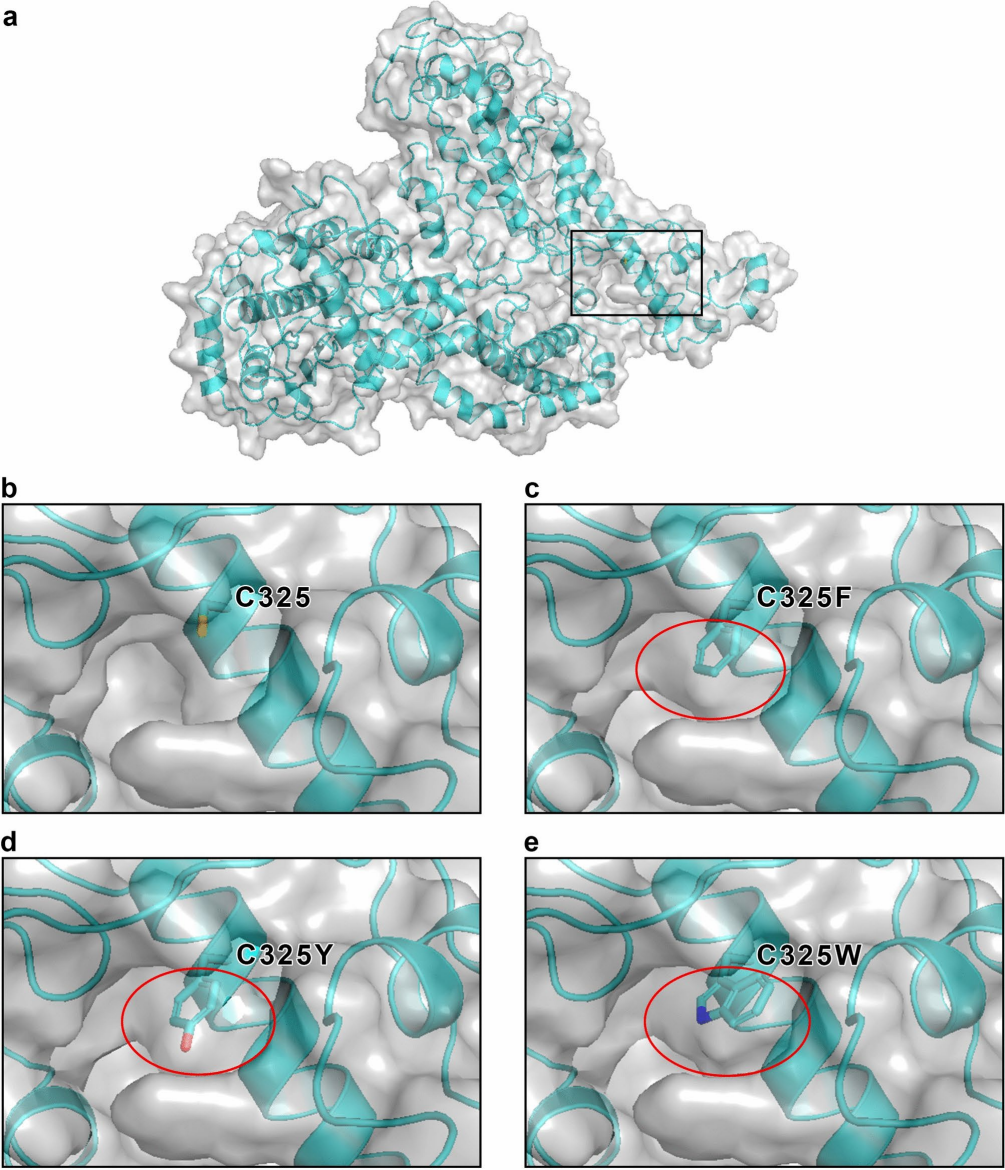
Reduction in cavity volume in C325 mutants compared to wild-type pUL56. (**a**) Localization of the pUL56 mutants. Shown is the cavity which has a reduced volume in the mutants. Reported cavity volume loss in comparison to the wild-type (**b**) was as: C325F: 129.38 Å^3^ (**c**), C325Y: 131.33 Å^3^ (**d**), C325W: 161.73 Å^3^ (**e**).

Letermovir is the first terminase inhibitor to be approved for prophylaxis against HCMV after hematopoietic stem cell transplantation. It has been suggested that letermovir prevents HCMV replication by binding to terminase subunits. A staggering number of described letermovir-induced mutants are in one region of pUL56 (Fig. 2a), suggesting a potential binding pocket for letermovir. For our analysis we used the most prominent letermovir mutants C325F, C325Y, C325W and V236M (Fig. 2b). To determine whether the mutants induce structural changes of pUL56 we performed analysis with the program Missense3D^33^. While the valine 236 mutant does not show any structural changes (Table 1) all three cysteine 325 variants lead to a pronounced cavity reduction compared to pUL56 wild type (Fig. 3). The mutant C325F reduced the cavity by 129.38 Å^3^, mutant C325Y by 131.33 Å^3^ and mutant C325W by 161.78 Å^3^ (Table 1). It should be noted that there are no other cysteine residuals at a distance of 10 Å to the C325 residual. Therefore, no loss of disulfide bonds is conferred by the studied mutations.

**Table 1:** Predicted structural changes introduced by mutations on pUL56.

Mutant	Structural change
C325F	cavity reduction by 129.38 Å^3^
C325Y	cavity reduction by 131.33 Å^3^
C325W	cavity reduction by 161.78 Å^3^
V236M	none

Predictions were made by Missense3D.

### Binding sites of letermovir in pUL56

**Fig. 4:**
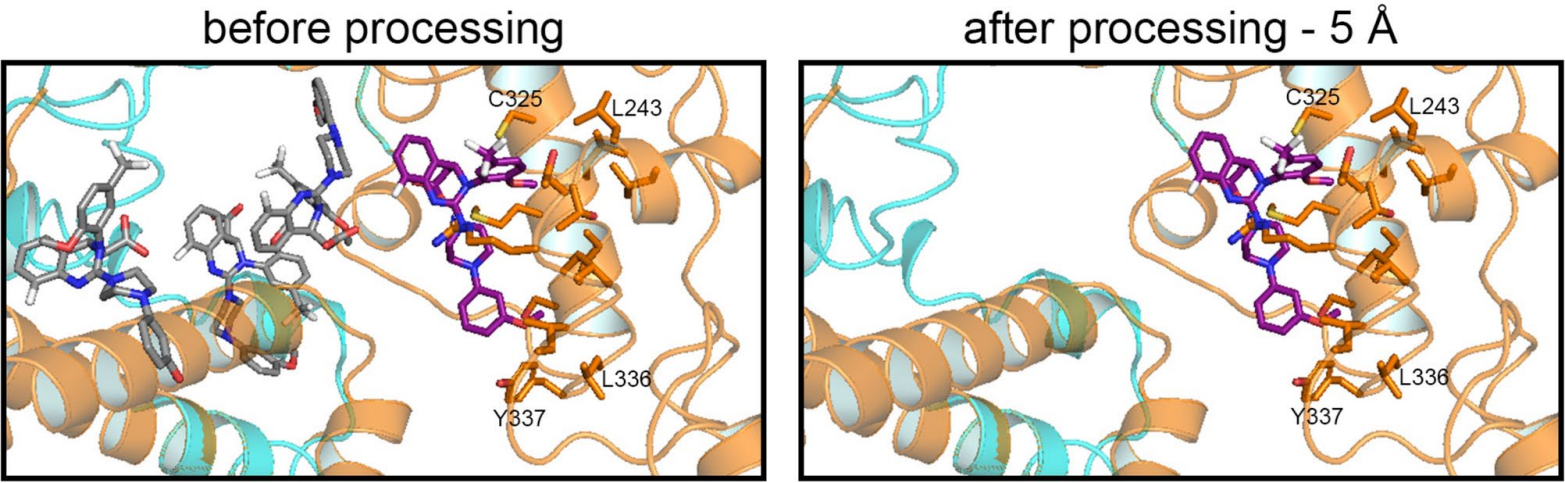
Data processing for letermovir binding modes within a 5 Å distance to region of interest. Before processing different binding sites of letermovir are shown (grey). Because some binding sides from SwissDock were not valid for this analysis, we filtered the binding modes based on specific criteria. We used PyMOLs build-in function modify—> around—> atoms within 5 A to determine whether the criteria is met. After processing, only the letermovir conformation within 5 Å of a defined set of amino acids of the potential binding pocket remained (violet). Amino acids used for filtering are shown as sticks and listed in Table S1. Oxygen-red, Nitrogen-blue, Fluor-white

To determine possible binding sites of letermovir, docking analysis with the program SwissDock^34^ was performed. The maximum number of docking sites output by the program is 256. To obtain the most probable conformations of letermovir, analysis was only performed with amino acids 1 to 99 and 223 to 383 of pUL56 (Figure S1). SwissDock computes many positions of a potential ligand. Some of these positions may be outside of the studied region or even on the outside of the protein itself. Therefore, the letermovir binding modes were processed to only include relevant conformations within a 5 Å range of defined amino acids protruding into the potential binding pocket (Table S1). This processing ensures that, for further analysis, only those letermovir conformations are considered which are able to interact with the potential binding site. The effect of letermovir conformation processing is exemplarily illustrated in Fig. 4.

### A letermovir binding pocket for the pUL56 wild type

**Fig. 5:**
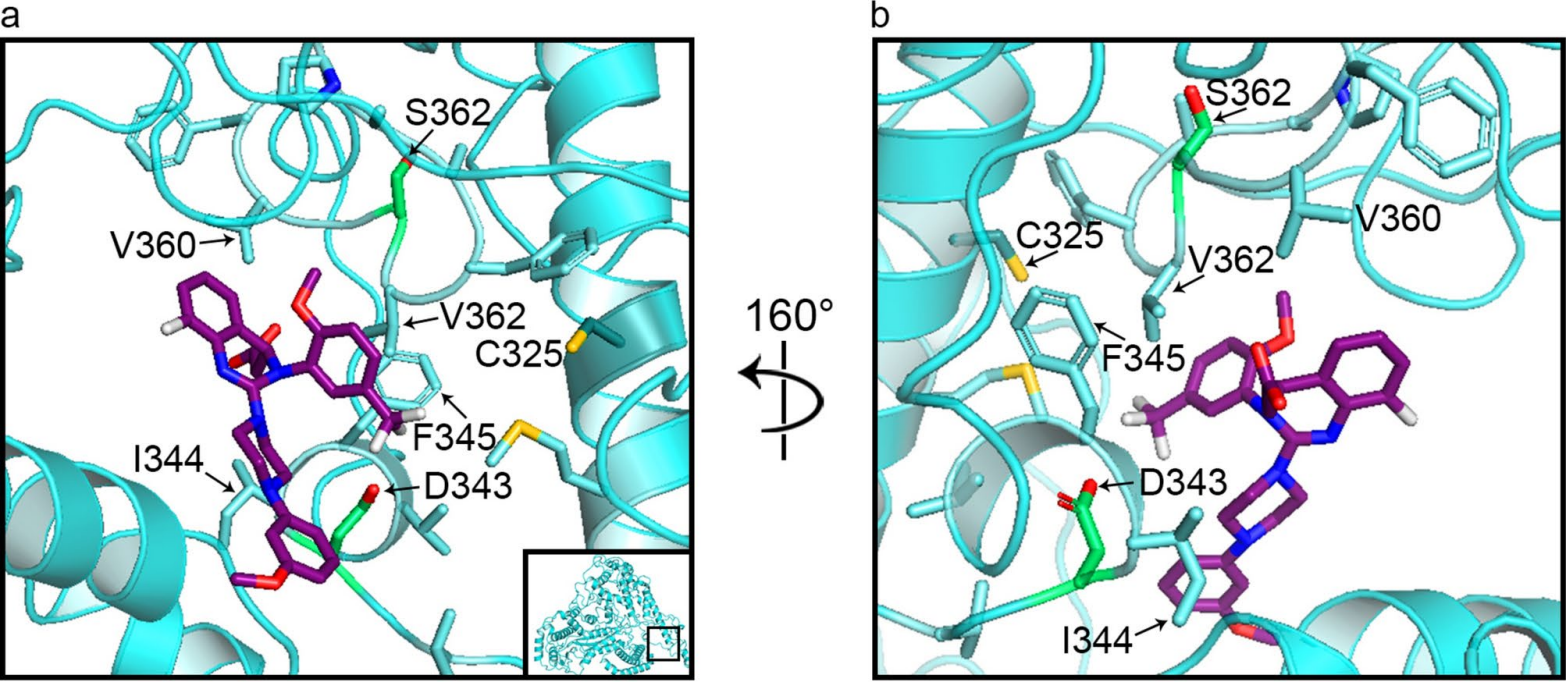
Binding of one relevant letermovir conformation to HCMV pUL56 wild type. The 15 most frequently interacting amino acids are shown as stick, with nonpolar amino acids in cyan and polar in green. The six most frequent amino acids are labeled. Note that C325 is shown for guidance, even though it is not interacting with the pUL56 wild type. The binding mode is shown at two different angles. Oxygen-red, Nitrogen-blue, Sulfur-orange, Fluor-white.

SwissDock returns 256 possible binding modes from every run. Here we analysed three independent runs for every pUL56 variant, leading to a total number of 768 letermovir conformations. After data processing as described in the method section, 318 relevant letermovir conformations remained for the pUL56 wild type. From the relevant letermovir conformations we analysed which 15 amino acids are most frequently interacting. Among these 15 most frequent amino acids are three polar amino acids and twelve nonpolar, shown in Table 2. It is also highlighted how many of the 318 cases involved a particular amino acid within 5 Å, indicating interaction with the letermovir molecule. Interestingly, the C325 residue is not among those 15 most frequently interacting amino acids, but is in contact with letermovir in the C325-mutants as shown below. Figure 5 shows the precise binding pocket of the pUL56 wild type predicted from our analysis. Letermovir is located in the center of the graphic (violet). The 15 most frequent amino acids from Table 2 are shown as sticks, with polar amino acids in green and nonpolar in cyan. The six amino acids with the highest occurrence are labelled in Fig. 5. Note that for visual representation the C325 residue is also shown in Fig. 5, even though it is not among the 15 most frequently interacting amino acids for the pUL56 wild type.

**Table 2:**
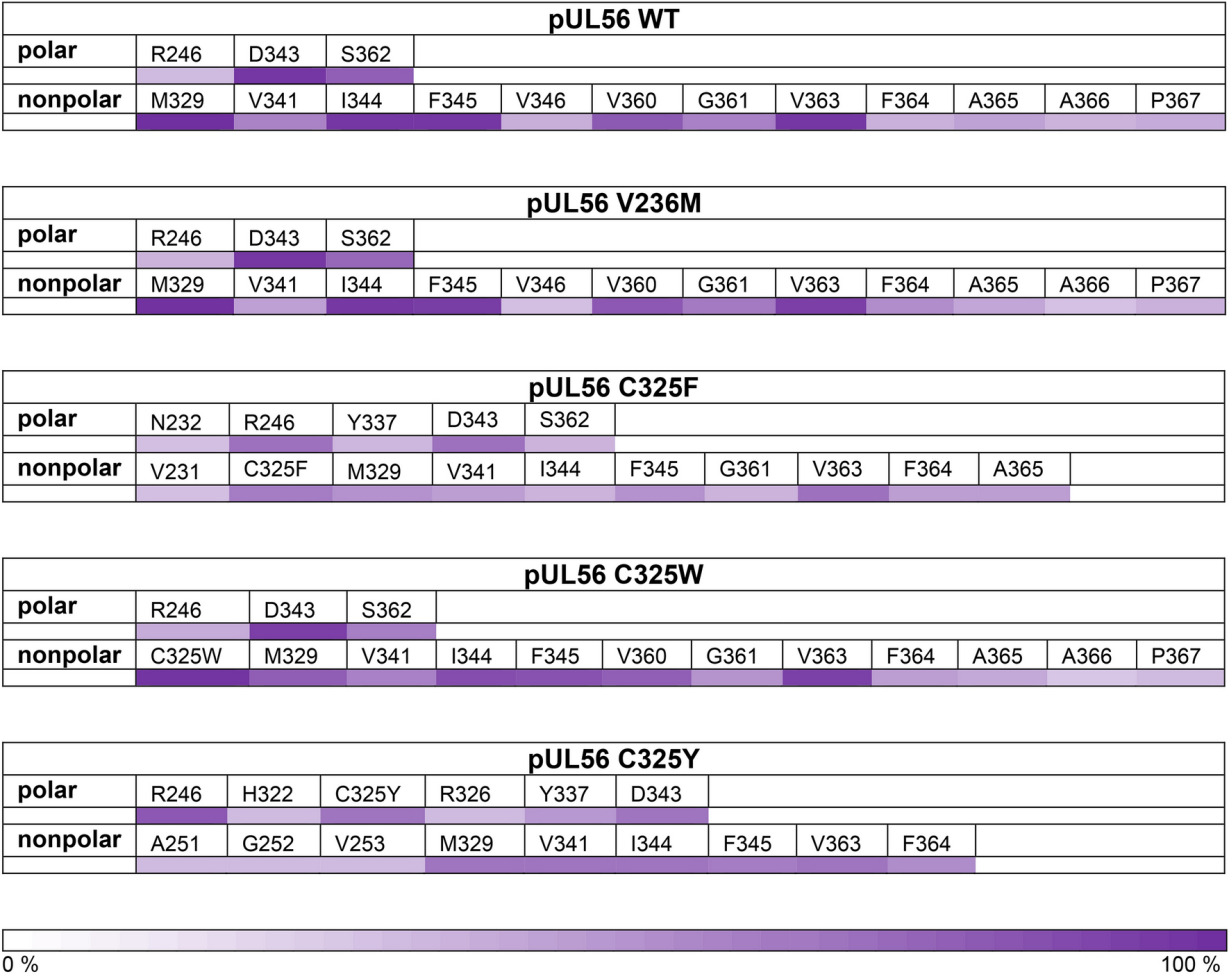
Amino acids contributing to the binding pocket of pUL56.

### Mutations in the C325 residue brings it into contact with letermovir

Based on binding analyses of the wild-type pUL56 protein and its substitution mutants, we identified the amino acid residues most frequently predicted to interact with letermovir.

**Fig. 6:**
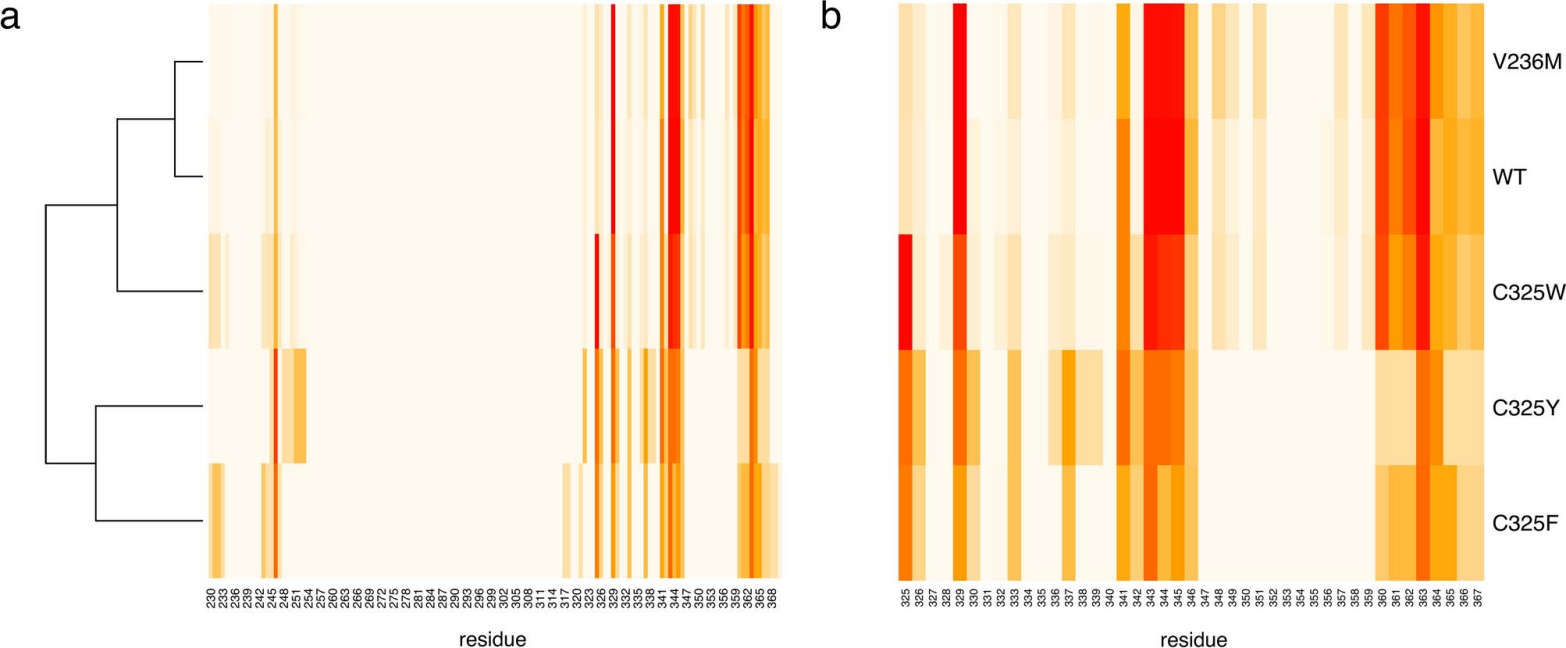
Binding profiles of each pUL56 mutant. Shown are heatmaps visualizing the binding profile of each pUL56 variant. A cell will have a darker color if the corresponding residue is in proximity to letermovir in a larger proportion of binding conformations of the pUL56 variant. In subfigure (**a**), this is shown across the entire putative binding site. In subfigure (**b**), a closer inspection of the region of residues 325–367 is shown, as the majority of letermovir-proximal residues lie here. Subfigure (**a**) additionally shows a hierarchical clustering of pUL56 variants based on letermovir-proximal residues.

Figure 6a shows the fraction of binding modes in which each amino acid residue (in the region of residues 230–370) was within 5 Å of letermovir, indicating interaction. The first domain of letermovir interaction predictions was between amino acids 326–329, follows by a second domain of interaction between amino acids 341–345 and a third domain between amino acids 359–363 (Fig. 6a). A hierarchical clustering based on letermovir—pUL56 variant interaction behavior found the variants C325F and C325Y to be most closely related (Fig. 6a). Interestingly, the C325W mutant shows more similarity to the wild type as to the other two C325-mutants. In Fig. 6b an enlargement of the area between amino acids 325–367 is shown. This analysis demonstrated that protein sections of amino acids 343–346 as well as amino acids 360–363 show the most common letermovir interactions in the putative binding site (Fig. 6b). The most remarkable finding here is that the C325 residual only come into contact with the letermovir molecule when this residual is mutated. This goes along with increased spatial area of the mutants and the cavity reduction seen in Fig. 3 and Table 1. For the C325F/Y/W mutations, the residual 325 belongs to the 15 most frequently interacting amino acids, which contrasts the behavior described above for the pUL56 wild type.

### Pairwise comparison between pUL56 variants and wild-type pUL56 ∆G values

A statistical analysis was performed to verify the significance of the obtained binding energies (∆G) of pUL56 wild type, pUL56 V236M, pUL56 C325F, pUL56 C325Y and pUL56 C235W (Table S2). Given the non-Gaussian and unbalanced nature of the data, a Kruskal–Wallis test was appropriately chosen to test the effect of mutation on binding affinities. The test showed a significant difference in ΔG values across the variants, indicating varying binding affinities (χ2 = 30.928, degrees of freedom = 4, p-value < 0.0001). Subsequent pairwise comparison by Wilcoxon rank-sum test found C325Y to have a significantly higher ΔG-value than the wild-type pUL56, thus indicating a lower affinity to letermovir (Table S2).

### Letermovir binding conformations are “pushed-out” of the binding pocket for C325F and C325Y

**Fig. 7:**
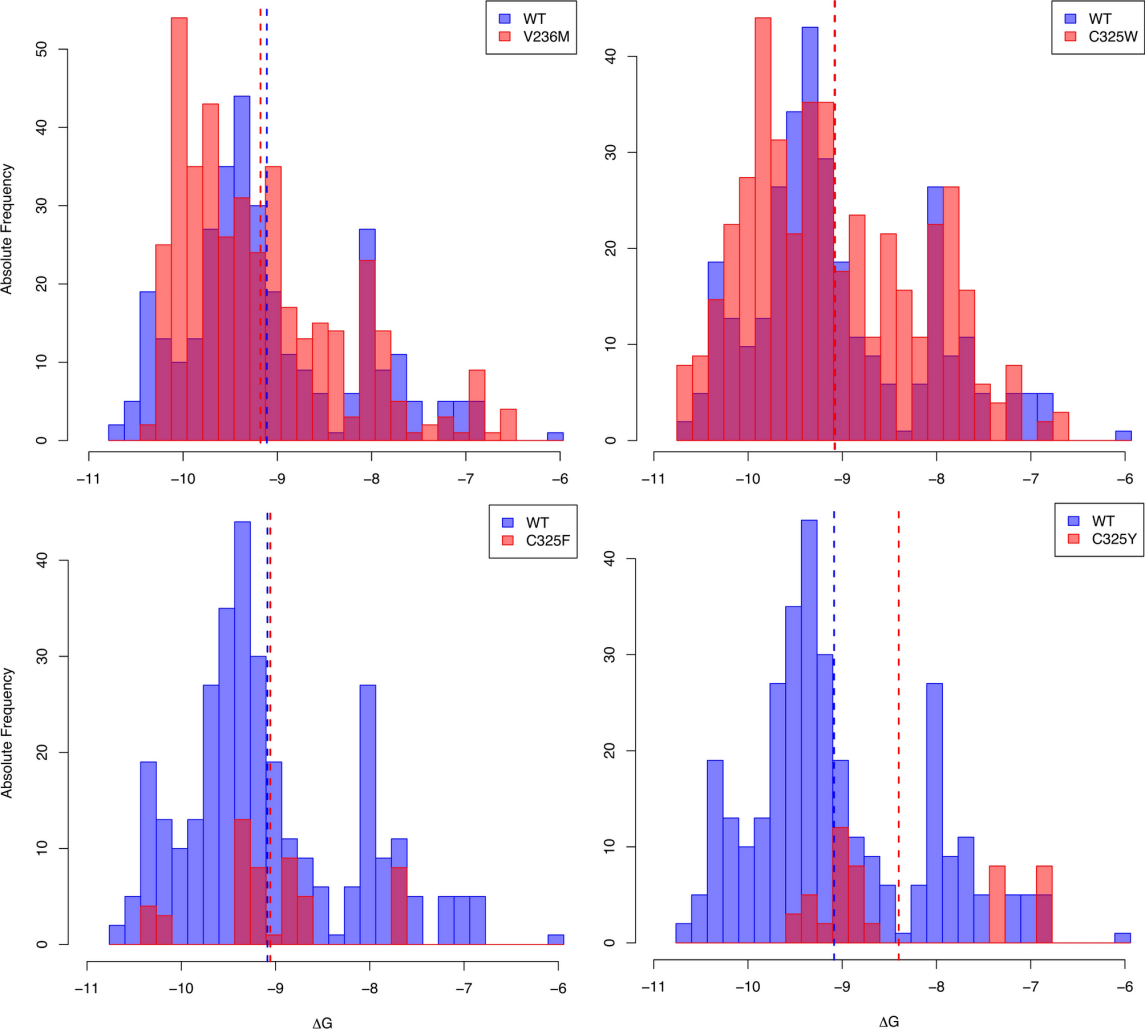
Distribution of the Gibbs free binding energy (∆G) for the pUL56 wild type (blue) compared to four different pUL56 mutants (red). Relevant conformations for each pUL56 variant are clustered into intervals and number of occurrences are counted (absolute frequency). For pUL56 V236M and C325W no change in the Gibbs free energy distribution is evident compared to the pUL56 wildtype, while the number of binding events are drastically reduced for pUL56 C325F and C325Y.

We compared the Gibbs free energy distributions of the pUL56 wild type, V236M, and C325 mutants by generating histograms of letermovir binding conformations. Energy values from relevant binding modes were grouped into intervals to visualize the frequency of conformations at different binding energies. Figure 7 shows this energy distribution with the wild type in blue and the mutant in red. For the V236M and the C325W mutant no difference compared to the wild type is evident (Fig. 7A, B). But strikingly, for the C325F and C325Y mutant, very few binding conformations remain after data processing (Table S2; Fig. 7C, D). This suggests that the letermovir binding modes are “pushed-out” of the binding pocket resulting in no possible binding for those mutants. This goes along with the steric hindrance from the pronounced cavity reduction shown in Fig. 3 and Table 1. Possible reasons for seeing no effect for the V236M and C325W mutant will be discussed below. Mean ΔG-values and number of viable binding modes for each pUL56 variant are found in Table S2.

## Discussion

A key step in replication of human cytomegalovirus (HCMV) in the host cell is the generation and packaging of unit-length genomes into preformed capsids. The enzymes involved in this process are the terminases. The HCMV terminase complex consists of two subunits, the ATPase pUL56 and the nuclease pUL89^13–16^. A potential third component pUL51 has been proposed to be involved in packaging^17,18^. Letermovir is the first terminase inhibitor approved by the FDA and European Medicines Agency for HCMV prophylaxis of hematopoietic stem cell recipients. It was predicted that letermovir prevents the formation of infectious viral particles by inhibiting the pUL56 activity^19^.

Despite the importance of letermovir and intensive research into biochemical aspects, there is still a lack of structural requirements leading to resistance. Here we focused on the drug target area in the HCMV terminase subunit pUL56. To gain further structural insights into the putative binding site of letermovir, in silico analysis were performed using Phyre2^27^. Although pUL56 has limited amino acid sequence identity with other herpesviruses it is commonly accepted that the structure–function relationship is homologous^35^. Therefore, accuracy of structure and function models are not affected by sequence identity. Recently the atomic structure of the herpes simplex virus type 1 terminase was solved^36^. Yang et al.^36^ showed that terminase subunit pUL28 is composed of six different domains according to their function in cleavage and packaging. By an overlay of the generated 3D structure of pUL56 with HSV-1 pUL28 (Ter^s^) it is obvious that it is highly consistent with the cryo-EM structure of pUL28.

The program SwissDock provides maximum 256 conformations of a ligand. To get the most letermovir binding modes inside the binding pocket, we only used the C-terminal part of pUL56 (Figure S1). It is known that amino acids side chains are very flexible. Therefore, setting a maximum distance to amino acids is a prerequisite. Mitchell et al.^37^ recommended that the distance between a ligand and a substrate should be five Ångstrom. A similar prediction was reported by Salentin et al.^38^. Therefore, only binding modes of letermovir within 5 Å of amino acid side chains of the binding pocket were included for further analysis. Overall, our results indicate that the majority of amino acids of the letermovir binding pocket are in the region of residues 230–370, the region described having the most letermovir induced pUL56 mutants^24,39,40^.

In recent years, many instances of resistance of letermovir in vitro and in vivo have been reported^41,42^. Here we analysed four pUL56 mutants that cover a wide range of effectiveness regarding the letermovir resistance. Those mutants are pUL56 V236M, pUL56 C325F, pUL56 C325Y and pUL56 C325W. Chou^24^ demonstrated that the degree of resistance corresponded to a highly increased EC_50_ ratio; e.g., EC_50_ of C325F/Y/W increases > 3000 fold and V236M increases up to 55-fold. An explanation for the less pronounced increase of V236M could be due to its strong similarity of interacting amino acids to the pUL56 wild type (Fig. 6). It is reasonable to assume that in nature conformational changes of pUL56 could arise while letermovir is present^43^. This may result in a closer proximity of V236M to the letermovir molecule, enabling it to interact with letermovir and increase the effect of this mutation. This could be one explanation why we did not see a mild change in the distribution of the Gibbs free binding energy for V236M compared to the wild type. However, since the EC_50_ ratio of the V236M mutant is magnitudes smaller compared to the C325-mutants, the V236M mutant could also be seen as a very weak mutation that is being expected to lead to almost no change in the energy distribution. We showed that pUL56 C325Y has the highest Gibbs free binding energy among the studied pUL56 variants, resulting in a weaker binding of letermovir. Pairwise comparison between the C325Y-mutant and the wild-type showed that this increase is statistically significant. Additionally, the distribution of the Gibbs free energy changed for the C325F and C325Y mutant with a drastic reduction of relevant binding conformations, suggesting that no binding can occur for these mutants. This goes along quite well with the observed added interaction of the C325 residual of the C325-mutants (Fig. 6) and the reduced cavity reduction (Fig. 3, Table 1). Even though the C325W mutant shows the largest cavity reduction, it might not affect the binding energy distribution, due to possibly changed nonpolar- or π-stacking-interactions with the letermovir molecule. Ultimately, in our analysis this mutant showed more similar letermovir binding behavior to the pUL56 wild type and the V236M mutant, than to the C325F and C325Y mutants (Fig. 6). However, it seems that cysteine at position 325 is a prerequisite of the letermovir binding. The reduced cavity for the C325-mutants as well as the observed “push-out” of the letermovir conformations from the binding pocket might be the reason that the mutants pUL56 C325F and pUL56 C325Y confers absolute resistance in vivo^44^. Regarding the mode of action of letermovir it was suggested that letermovir might interfere with the pUL56 DNA binding activity. This was reasoned by the presence of a LAGLIDADG (endonuclease) and a zinc finger motif—two DNA recognizing patterns—in the sequence proximity of known mutations^32^. This goes along with the reported loss of correctly cleaved viral DNA and the reduction of infectious particles when letermovir is present^19^. Additionally, we would like to note that for isolated pUL56 we saw a reduced ATPase activity under letermovir treatment (unpublished data). However, in our three-dimensional model of pUL56 neither the DNA binding motifs nor the ATP binding domain^30^ is spatially near to the region of know mutations and the here proposed letermovir binding pocket. This could indicate that binding of letermovir to pUL56 might elicit global conformational changes in pUL56. These conformational changes could then result in multiple modes of actions like prevention of DNA binding/cleavage, reduction in ATPase activity and/or destabilization of the terminase complex. Similarly, this was shown for the benzimidazole-D ribonucleoside Cl_4_RB—another HCMV terminase inhibitor. Cl_4_RB prevents the interaction of pUL56 with the pUL104 portal domain, as well as DNA cleavage^45^. Therefore, we propose that letermovir might be an allosteric inhibitor, mediating its inhibitory function through conformational changes in pUL56. The data presented here provides new insights on the mode of action and the binding site of letermovir.

In summary, we computed the first three-dimensional structure of pUL56 from HCMV. Furthermore, our data offers new insights into most frequent mutation during high letermovir treatment, C325F and C325Y, covering a wide range in their anti-drug effectiveness. We identified a potential binding pocket of letermovir in the region aa240-aa337 that leads to further information about its inhibitory function, which we suspect to be allosteric. According to our results the structure of letermovir might be modified to circumvent resistance.

## Material and methods

### Modelling of pUL56

Structure predictions were carried out with the Phyre2 web portal for protein modelling, prediction, and analysis (in intensive mode)^27^. This program created in the first step a hidden Markov model (HMM) that is then compared against a database of HHMs of proteins with known structure, including homologous proteins from bacteriophage and herpesviruses (e.g., HSV-1 pUL28). The resulting first models are subject to further stepwise analysis to optimize the structure of pUL56^27^. For modelling and generating pUL56 mutants, the program PyMOL (The PyMOL Molecular Graphics System, Version 2.0, Schrödinger, LLC) was used.

### Ligand binding via SwissDock

To identify potential binding sites of letermovir, in silico analysis with SwissDock^34^ (Swiss Institute of Bioinformatics, Lausanne) was performed. SwissDock is a web service for predicting the interactions that may occur between a target protein (pUL56) and its ligand (letermovir). Prior to docking, the generated 3D structure of pUL56 was optimized by the H +  + Server^46^, a tool that automates the process to quickly obtain the protonation states of amino acids residues at a given pH value. Since pUL56 is localized in the nucleus^24^ the pH was defined as 7.2. The optimized 3D structure of pUL56 and the structural model of letermovir (ZINC database^47^) were used for the analysis. SwissDock computes the binding affinity of a given letermovir conformation in interaction with the amino acid side chains of the protein. In addition, SwissDock provides a built-in option that takes the flexibility of the amino acid side chains into account. In our analysis, the amino acid side chains were flexible in a radius of 5 Å around the letermovir molecule^37^. The output of SwissDock are the conformations of the ligand as well as the corresponding Gibbs free binding energy (∆G). Additionally, the SwissDock data was processed for relevant letermovir conformations. For this, only conformations remained that were within a 5 Å distance to set of defined amino acids (Table S1, Fig. 3). To utilize this, we programmed a python script which makes use of the PyMOL build-in function “modify—> around—> atoms within 5 A” to select the corresponding amino acids around the letermovir molecule. This was also used to identify the amino acids of the potential binding pocket within a radius of 5 Å to letermovir. The corresponding amino acids were selected and visualized.

### Analysis of frequently interacting residues

To characterize the binding motif proposed by SwissDock, we identified the residues that most frequently interacted with letermovir. For this, a heatmap was generated showing pUL56 variants as rows and pUL56 residues as columns. Each cell in the heatmap illustrates in what fraction of binding motifs of the pUL56 variant the given residue is in proximity to letermovir. A hierarchical clustering of pUL56 variants was also done to find out which variants showed similar letermovir binding profiles.

### Statistical analysis

Analysis of the filtered binding motifs was conducted using base R version 4.3.0. To assess the distribution of the data, a Shapiro–Wilk test was applied for testing normality. For comparing the ΔG-values, which were used to quantify the association strength between letermovir and the various pUL56 variants, a Kruskal–Wallis test was employed. Pairwise comparisons of variants were done using a Wilcoxon rank-sum test and Holm’s method^48^ used for correction of *p*-values for multiple testing.

## Supplementary Information

Supplementary Information.

## Data Availability

The raw data will be stored on an electronic data processing medium for 10 years (according to the institute’s guidelines for laboratories). All other data will be stored by the IT service on the server of the Charité Universitätsmedizin Berlin. All data are available on request to the corresponding author.
