# Supplementary material for: Identification of a binding pocket of letermovir in the terminase subunit pUL56 of human cytomegalovirus
Source: Sci Rep. 2025 Mar 25;15:10334. doi: 10.1038/s41598-025-94809-1 (PMC11937570; doi:10.1038/s41598-025-94809-1)
Supplement: Supplementary file 1 — Supplementary Information. [file 41598_2025_94809_MOESM1_ESM.pdf]

Supplement materials

Identification of a binding pocket of Letermovir in the terminase subunit pUL56 of human Cytomegalovirus

Lukas M. Kmetsch, Hans Tietze , and Elke Bogner

Fig. 1Supp
Kmetsch et al.

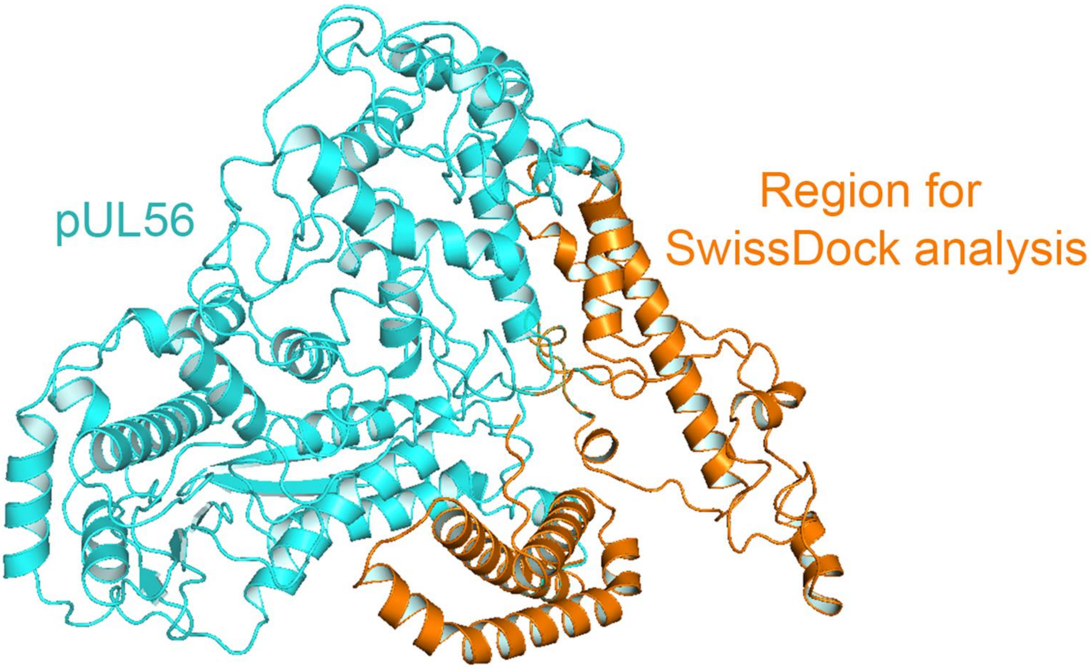

Figure S1. Model of the 3D structure of pUL56 with indicated regions for data processing.

Table S1. Amino acids for processing

Amino acids for processing SwissDock conformations									
**polar**	T244	T245	R246	S333	Y337				
**nonpolar**	L240	L243	I247	L250	C325	L238	M329	I332	L336

Table S2: Mean values of Gibb's free energy in kcal/mol for each pUL56 variant.

**pUL56 Variant**	**n_{BM}**	**ΔG_{mean} in kcal/mol**	**p-value, pwc with wt**	**significance**
Wildtype	318	-9.09 ± 0.91	-	-
V236M	400	-9.15 ± 0.86	0.72	
C325W	445	-9.06 ± 0.75	0.95	
C325F	51	-9.09 ± 0.91	0.72	
C325Y	48	-8.40 ± 0.96	<0.0001	***

($p < 0.05$: *, $p < 0.01$: **, $p < 0.001$: ***). Correction of p-values was done using Holm's method.

ΔG-values were directly obtained by SwissDock for each Letermovir binding mode. ΔG-values were averaged by the remaining conformation after processing. Number of binding modes remaining after processing are given as n_{BM}. The "p-value, pwc with wt" column notes the p-value resulting from a pairwise comparison (pwc) between mutant and wild-type (wt).
